# Group-based Education and monitoring program delivered by community health workers to improve control of high blood pressure in island districts of lake victoria, Uganda

**DOI:** 10.1186/s12875-024-02444-y

**Published:** 2024-05-28

**Authors:** Andrew Kwiringira, Richard Migisha, Lilian Bulage, Benon Kwesiga, Daniel Kadobera, George Upenytho, Paul Mbaka, Julie R. Harris, Donald Hayes, Alex R. Ario

**Affiliations:** 1 Uganda Public Health Fellowship Program, Kampala, Uganda; 2 Uganda National Institute of Public Health, Kampala, Uganda; 3 Department of Community Health, Ministry of Health, Kampala, Uganda; 4 Department of Planning Financing and Policy, Ministry of Health, Kampala, Uganda; 5 US Centers for Disease Control and Prevention, Kampala, Uganda; 6 Division of Heart Disease and Stroke Prevention, US Centers for Disease Control and Prevention, Atlanta, GA USA

**Keywords:** Health Education, Hypertension, Community Health workers, Uganda

## Abstract

**Background:**

Individuals living in communities with poor access to healthcare may be unaware of their high blood pressure (BP). While the use of community health workers (CHWs) can address gaps in human resources for health, CHWs in Uganda have not been used previously for BP screening and management. We report the results of an initiative to train CHWs to evaluate BP and to administer group-based education in Kalangala and Buvuma Island Districts of Lake Victoria, Uganda.

**Methods:**

We randomly selected 42 of 212 villages. We trained CHWs based in island districts on measuring BP. CHWs visited all households in the selected villages and invited all adults ≥ 18 years to be screened for high BP. We used the World Health Organization’s STEPwise tool to collect data on demographic and behavioral characteristics and BP measurements. High blood pressure was defined as systolic BP (SBP) ≥ 140 mm Hg and/or diastolic BP (DBP) ≥ 90 mm Hg over three readings. CHWs created and led fortnight support groups for individuals identified with high blood pressure at baseline. At each group meeting, CHWs re-measured BP and administered an intervention package, which included self-management and lifestyle education to participants. The paired t-test was used to compare mean values of systolic blood pressure (SBP) and diastolic blood pressure (DBP) before and after the intervention. Generalized estimating equations (GEE) were used to model longitudinal changes in BP.

**Results:**

We trained 84 CHWs to measure BP and deliver the intervention package. Among 2,016 community members, 570 (28.3%) had high blood pressure; of these, 63 (11.1%) had a previous diagnosis of hypertension. The comparison of SBP and DBP before and after the intervention revealed significant reductions in mean SBP from 158mmHg (SD = 29.8) to 149 mmHg (SD = 29.8) (*p* < 0.001) and mean DBP from 97mmHg (SD = 14.3) to 92mmHg (*p* < 0.001). GEE showed decreases of -1.133 (SBP) and − 0.543 mmHg (DBP)/fortnight.

**Conclusion:**

High BP was common but previously undiagnosed. The CHW-led group-based self-management and education for controlling high BP was effective in the island districts in Uganda. Scaling up the intervention in other hard-to-reach districts could improve control of high BP on a large scale.

## Introduction

Hypertension (high blood pressure) is a leading contributor to the global burden of disease and premature death, accounting for approximately 9.4 million deaths annually [1]. As of 2015, the prevalence of hypertension was 22.2% among adults aged 18 to 64 years in Uganda; however, 77.6% had hypertension that was not previously diagnosed [2]. This highlights a gap in underdiagnosis among the population. When left untreated or inadequately managed, hypertension can result in potential complications and increased risk of cardiovascular diseases, strokes, and other related health issues [3]. The prevalence of undiagnosed high blood pressure is higher in areas where there is limited access to healthcare facilities and essential medicines [4], such as the island districts of Lake Victoria, compared to areas with better access to healthcare. Most households in these island districts live beyond the targeted five kilometer’s maximum from the nearest health facility [5].

The use of community health workers (CHWs) is one strategy to address inaccessibility to healthcare facilities and gaps in human resources for health [6]. CHWs are frontline public health workers and aides selected, trained, and working in the communities from which they come. They perform a variety of tasks such as home visits, sanitation, first aid, maternal and child health and family planning activities. However, the role of CHWs and their contributions in many countries, including Uganda, is largely focused on maternal and child health as well as the prevention and treatment of infectious diseases [7]. This underscores a gap in leveraging CHWs to address non-communicable diseases such as hypertension. Although some studies have shown the effectiveness of CHWs in management of hypertension using individual-based interventions [8–10], these studies were intensive, costly, highly structured, and time-consuming. This raises concerns about scalability and sustainability, particularly in resource-constrained settings like Uganda.

Population-level public health measures, such as high blood pressure screening and health education and lifestyle modifications like diet and exercise, are generally considered more cost-effective means of controlling hypertension than treatment-oriented programs like prescription medication [11], which most developing countries lack the capacity to implement on a large scale [12]. Our approach of using a CHW-led group-based education and blood pressure monitoring program to early diagnose and prevent chronic hypertensionbridges gaps in healthcare access in hard to reach regions. By empowering CHWs to conduct group-based education and blood pressure monitoring, we aim to enhance early diagnosis in populations that may otherwise face significant barriers to healthcare services.

This project incorporated comprehensive data collection at baseline and during the intervention which enabled us to assess the effect of the intervention and to assess the strength and challenges of implementing the program. We determined whether a CHW-led group-based education and monitoring program for the management of high blood pressure is effective in a hard-to-reach setting.

## Methods

### Study design

The project employed a pre-post intervention design without a comparison group. Initially, a baseline community-based survey was conducted by CHWs to identify participants with high blood pressure, followed by a CHW-led group-based education and blood pressure monitoring intervention administered to participants identified to have high blood pressure at baseline.

### Project setting and population

The project was conducted in two island districts of Lake Victoria: Buvuma and Kalangala, neither of which have inland territory. As of 2023, the population of Kalangala District was 74,500, while Buvuma District’s population was 154,200. Both island don’t have territory on mainland Uganda, fishing and subsistence agriculture serve as the primary economic activities [13]. Most households in these island districts live beyond the targeted five kilometer’s maximum from the nearest health facility [5]. Health facilities are also inadequately equipped to provide screening and management of hypertension [14], and health workers are not always available in these settings [15]. Population surveys have provided hypertension prevalence data at the regional level but not at the district level. Additionally, routine Health Management Information System (HMIS) data does not distinguish between known cases of hypertension and new cases, making it inadequate for accurately estimating prevalence. In each district, we employed random selection methods to choose one parish from each of the 7 sub-counties and three villages from each selected parish. All chosen villages were included in the cross-sectional survey.

### Sample size

We employed the sample size formula for paired samples. Specifically, we aimed to detect a mean difference of 6 mmHg in systolic blood pressure and 2 mmHg in diastolic blood pressure from baseline to endline, with a desired power of 80% and a significance level of 0.05. We incorporated the Z-values corresponding to the desired significance level and power, along with the estimated standard deviation of the measurements. As a result, we determined that a sample size of 296 individuals with high blood pressure was necessary to achieve adequate statistical power to detect the specified changes in blood pressure levels.

### Intervention

Prior to beginning the study, 84 CHWs were trained during a 5-day course to measure BP and deliver group-based self-management and education. *The Ministry of Health (MOH) village health team trainers employed a training approach for community health workers (CHWs), incorporating theoretical, practical, and interactive components. The theoretical sessions covered an overview of high blood pressure, including its causes, risk factors, and potential complications. Practical skills development involved hands-on training sessions where CHWs practiced measuring blood pressure on simulated patients under trainer supervision.* Additionally, CHWs practiced delivering lifestyle modification counseling and motivational interviewing techniques. Training sessions were conducted from the selected community centers. We trained all CHWs who were already working in the selected villages. The training focused on education about high blood pressure and how to manage it, adherence to medications, and lifestyle changes, such as increasing physical activity and following a healthier diet. CHWs then invited all residents ≥ 18 years to participate in the cross-sectional survey. CHWs conducted house-to-house notification and encouragement to ensure that all residents ≥ 18 years were informed about the survey. CHWs approached participants at their homes and provided a written participant information sheet. We used the WHO STEPS protocol to collect data on demographic and behavioral characteristics (alcohol use, tobacco use, physical inactivity)and BP measurements [16]. CHWs measured blood pressure (BP) after the participant had been seated quietly for at least 15 min. Three measurements were taken at 3-minute intervals using the appropriate cuff size and a calibrated digital automatic BP monitor according to the WHO STEPS protocol [25]. All participants with systolic BP (SBP) ≥ 140 mm Hg and/or ≥ diastolic BP (DBP) 90 mm Hg over three readings were informed they may have high blood pressure and were advised to visit a clinician to have their BP re-checked.

CHWs created and led group-based self-management and education support groups of up to 20 individuals identified with high blood pressure at baseline. These group meetings, each lasting ~ 90 min, were held every 2 weeks for 3 months within the villages in which the participants resided. No incentive was given to the participants to join the group meetings. At each group meeting, CHWs re-measured BP and delivered education about high blood pressure and how to manage it, in the local language. This included details about adhering to medications and the importance of lifestyle changes, such as increasing physical activity and following a healthier diet. Pictorial flipcharts were used as education aids, and handouts about high blood pressure control were provided to participants to use at home. CHW supervisors attended group training sessions to ensure standardized training across all groups, thereby minimizing potential variations in intervention delivery.

Four focus group discussions (FGDs) were conducted with CHWs and eight FGDs with residents who attended the group meetings among the two districts. In Buvuma District, this included two FGDs with an average of 9 CHWs and four FGDs with an average of 11 residents. In Kalangala District, two FGDs were conducted with an average of 9 CHWs and four FGDs with an average of 10 residents. FGDs were conducted with CHWs to explore the experiences, strengths, and challenges of implementing the intervention. In addition, FGDs were held with residents to learn more about the difficulties in diagnosing, treating, and controlling high blood pressure. The participants for the FGDs were selected by the research team to include a diverse range of individuals from various demographic backgrounds. Data collection for the FGDs was carried out by trained facilitators who were members of the research team.

### Ethical consideration

These facilitators received training on FGD moderation techniques, ethical considerations, and data handling procedures to ensure consistency and reliability across sessions. While audio recording was employed to capture the discussions accurately, participants were assured of confidentiality, and their identities were anonymized to maintain privacy and confidentiality throughout the research process.

### Outcomes

In this study, the primary outcomes included the prevalence, awareness and riskfactors of high blood pressure in the community, change in SBP and DBP from baseline, measured in accordance with the WHO STEPS protocol [25]. The secondary outcomes included the CHWs experiences, challenges to implementing the intervention, and participants’ perceptions of the support obtained from the CHWs and their experiences in managing their high blood pressure.

### Data analysis

We analyzed data in STATA version 16 (Statcorp, College Station, TX, USA). Prevalence of high blood pressure was reported as a proportion estimated in STATA. We used modified Poisson regression to assess factors associated with high blood pressure. We utilized the log link function in the modified Poisson regression model to estimate prevalence ratios (PRs) and their corresponding confidence intervals for the association between the explanatory variables and the outcome of interest. To account for this clustering, we utilized robust standard errors in our analysis. Robust standard errors were calculated to provide more accurate estimates.

We used the unpaired t-test to compare the mean SBP and DBP before and after the intervention. Generalized estimating equations (GEE) were used to model correlated and longitudinal data for investigating the longitudinal changes in BP after controlling for possible confounding factors (age, sex, tobacco use, and alcohol use) and accounting for clustering effects. Factors with *P* values ≤ 0.05 were considered as significant. Thematic analysis of focus group discussions was used to investigate the mechanisms of impact. FGDs were transcribed verbatim, translated back into English, and coded inductively using NVivo version 10 software. We used a stage-by-stage procedure to identify emerging themes, which were classified and combined to get more relevant, coherent, and inclusive themes. Attention was paid to the most recurring ideas and expressions as constituting our major themes.

## Results

### Characteristics of participants

Of the 2,016 participants, 1,201 (59.6%) were female, 1,076 (53.3%) were aged between 20 and 39 years, and 857 (43.4%) had attained at least secondary school education (Table 1).

**Table 1:** Characteristics of participants, effectiveness of CHW-led group-based education and monitoring program in island districts of Lake Victoria, Uganda, 2021 (*N* = 2016)

Characteristic	*N*	%
**Age**		
18–19	180	8.9
20–29	658	32.6
30–39	418	20.7
40–49	384	19.0
> 50	376	18.7
**Sex**		
Female	1,201	59.6
Male	815	40.6
**Education**		
Primary	774	38.4
None	367	18.2
Secondary	624	30.9
University	251	12.5
**Alcohol use**		
Yes	575	28.5
No	1441	71.5
**Tobacco use**		
Yes	109	5.4
No	1907	94.6

### Prevalence of high blood pressure

Of the 2,016 participants, 570 (28.3%) had high blood pressure; of these, only 63 (11.1%) knew that they had a previous diagnosis of hypertension.

Compared to participants aged 18–19 years, the unadjusted prevalence ratio (uPR) of high blood pressure was higher among persons aged 40–49 years (uPR = 2.5; 95% CI = 1.2–5.3) and ≥ 50 years (uPR = 3.5; 95% CI = 1.7–7.1) (Table 2). The prevalence of high blood pressure was not significantly different between males and females (uPR = 0.9; 95% CI = 0.7–1.1). In addition, there was no significant difference in prevalence of high blood pressure by level of education, tobacco use, and alcohol use.

**Table 2:** Factors associated with high blood pressure, effectiveness of CHW-led group-based education and monitoring program in island districts of Lake Victoria, Uganda, 2021

Variable	High blood pressure Percent	High blood pressure		Unadjusted PR (95% C.I)	*p*-value	Adjusted PR* (95% C.I)	*p*-value
		Yes	No				
**Age**							
18–19	13.9	25	155	1.00			
20–29	20.4	134	524	1.46 (0.96–1.95)	0.10	1.40 (0.90–1.91)	0.48
30–39	27.5	115	303	1.51 (1.01–2.15)	0.90	1.53 (1.03–2.03)	1.00
40–49	35.7	137	247	2.50 (1.24–5.30)	0.01	2.65 (1.29–5.46)	0.01
> 50	41.2	155	221	3.51 (1.74–7.10)	< 0.001	3.61 (1.79–7.29)	< 0.001
**Sex**							
Female	28.1	337	864	1.00			
Male	28.6	233	582	0.88 (0.69–1.11)	1.17	1.10 (0.91–1.23)	0.48
**Education**							
Primary	29.8	231	543	1.00			
None	28.9	106	261	0.97 (0.80–1.17)	0.74	0.96 (0.79–1.17)	0.69
Secondary	27.9	174	450	0.93 (0.79–1.10)	0.42	1.01 (0.85–1.18)	0.95
University	23.5	59	192	0.79 (0.61-1.00)	0.06	0.83 (0.65–1.06)	0.13
**Alcohol use**							
Yes	29.4	169	406	1.00			
No	27.8	401	1,039	0.95 (0.81–1.10)	0.49	0.96 (0.82–1.11)	0.56
**Tobacco use**							
Yes	31.2	34	75	1.00			
No	28.1	536	1,371	0.90 (0.67–1.20)	0.48	1.06 (0.77–1.47)	0.70

### Factors associated with high blood pressure

Old age was associated with high blood pressure. The prevalence of high blood pressure was significantly higher among persons aged 40–49 years (aPR = 2.7; 95% CI = 1.3–5.5) and ≥ 50 years (aPR = 3.6; 95% CI = 1.8–7.3) compared to participants aged 18–19 years. Sex, level of education, tobacco use, and alcohol use were not associated with high blood pressure (Table 3).

**Table 3:** Mean systolic and diastolic blood pressure before and after a 3-month CHW-led group-based education and monitoring program in island districts of Lake Victoria, Uganda, 2021

Variable	Before	After	Difference	*P*-value
SBP	159 ± 29.8	149 ± 29.8	9	< 0.001
DBP	97 ± 14.3	92 ± 14.3	5	< 0.001

SBP: Systolic blood pressure; DBP: Diastolic blood pressure; *P*-value: Probability value

### Effect of the intervention on high blood pressure control

Out of 570 participants identified with high blood pressure at baseline, 552 (97%) were included in the intervention program. Out of 18 participants who declined to participate in the intervention, 12 frequently travelled out of their districts, and 6 were immobilized at their homes. Overall, approximately 15 participants attended each of the meetings. Overall, each participant attended at least 5 meetings.

The comparison of systolic blood pressure (SBP) and diastolic blood pressure (DBP) before and after the intervention revealed significant reductions in mean SBP from 158mmHg (SD = 29.8) to 149 mmHg (SD = 29.8)(*p* < 0.001) and mean DBP from 97mmHg (SD = 14.3) to 92mmHg (*p* < 0.001) following 3-months of intervention (Table 3). After adjusting for confounding variables: age, sex, alcohol use, tobacco use, and GEE, the SBP, and DBP decreased by 1.13 (SE = 0.035) and 0.54 (SE = 0.028) mmHg/fortnight, respectively. *Unadjusted analysis revealed a reduction of -1.484 mmHg (SD = 0.731, p < 0.001) in SBP. After adjusting for age, sex, alcohol use, tobacco use and clustering within communities, the effect size remained significant, with a decrease of -1.133 mmHg (SD = 0.682, p = 0.001). DBP also exhibited a decrease in both unadjusted and adjusted analyses. The unadjusted analysis showed a reduction of -0.695 mmHg (SD = 0.362, p < 0.001) in DBP. After adjustment, the effect size was − 0.543 mmHg (SD = 0.264, p = 0.010)* (Table 4).

**Table 4:** The effect of the intervention on changes in blood pressure, CHW-led group-based education and monitoring program in island districts of Lake Victoria, Uganda

Unadjusted Results			Adjusted^*^ Results	
	β (SD)	*p*-value	β (SD)	*p*-value
Systolic BP	-1.484 (0.731)	< 0.001	-1.133 (0.682)	< 0.001
Diastolic BP	-0.695 (0.362)	< 0.001	-0.543 (0.264)	< 0.010

*adjusted for clustering

### Resident participant experiences with the intervention program

The most common motivation for participants to attend the meetings was to learn more about high blood pressure and how to manage it. Participants stated that the information they received from their healthcare providers about how to control hypertension was insufficient, consisting mostly of prescriptions for medication.

*“I attended group meetings to learn about high blood pressure, our health workers don’t give us enough information when we go to clinics, they write drugs and only tell you how to swallow them.” — Participant*.

Participants stated that the meetings provided them with knowledge about high blood pressure and how to manage it through simple and practical lifestyle changes such as diet, exercise, and medication adherence.

*“I got knowledge on high blood pressure, mainly knowledge on how to control it, and we used simple behaviour change approaches like diet, physical exercise, and drug adherence.” — Participant*.

*“The sessions provided me with practical knowledge on how simple lifestyle changes, like reducing salt intake, can make a big difference.” -Participant*.

The lack of medication distribution during the meetings was a major topic of discussion among the participants. Participants expected medications to be distributed and they expressed their belief that providing medications would increase attendance at meetings. Participants reported that the high cost of medication was a barrier to maintaining BP control.

*“We thought we were going to be getting high blood pressure drugs in our group meetings but we didn’t, attendance can be high if drugs are given” — Participant*.

Participants who attended meetings suggested that the program should be improved by providing medications during meetings, including free blood sugar monitoring, home visits, reminders to attend meetings, and ensuring convenient meeting locations in addition to blood pressure monitoring.

Participants expressed their satisfaction with the program. Participants also reported the positive impact of the CHW program on improving access to medical services and understanding of the high blood pressure control.

*“Previously, transportation posed challenges for me, making it tough to attend medical appointments. However, the CHW has been supportive and they are easy to access because they live in our commuinty.” -Participant*.

*“I’ve gained a deeper understanding of how to control high blood pressure by doing regular physical activity and reducing the amount of salt that I eat.”*

### Community health workers’ experiences, strengths, and challenges of implementing the intervention

CHWs are appreciated and esteemed within their communities for the support and care they provide. CHWs also expressed a strong collaborative bond with healthcare providers, acknowledging the positive acknowledgment of their contributions by health workers.

*“In our community, the work we do as CHWs is not just seen as a job. People look up to us for guidance and support, and that appreciation means everything to us.” — CHW.*

*“Healthcare providers recognize that our presence eases their workload. If you visit the outpatient department, you’ll notice shorter queues compared to before. This is because we assist people in the villages through home visits.” — CHW.*

Community Health Workers value a supervision framework in which they report to their designated CHW supervisors. They have articulated that this structure, alongside mentorship and supportive oversight, facilitated the transfer of knowledge and skills, enhanced task performance, and bolsters the program’s effectiveness.

*“When faced with an issue, I communicate it to my supervisor to seek assistance… The supervisors provide guidance on how to engage with households that may pose challenges or seem inaccessible.“-CHW*.

The CHWs face challenges in transportation. Certain villages and areas consist of households situated far apart from each other. As a result, CHWs encounter mobility issues despite having reasonable household allocations, which in turn affect their performance.

*“We have to traverse long distances to reach households within our villages. In my situation, there are days when I work tirelessly just to visit three households due to the long distance between them.“-CHW*.

CHWs expressed their support for the meetings and the importance of continuing them in their communities. Participants suggested that future meetings could benefit from the provision of blood pressure medications and the incorporation of home visits. CHWsalso suggested that future meetings would benefit from including previous participants. CHWs also reported that training, and supportive supervision of them during the intervention were important enablers of intervention implementation.

*“We should include our participants who are self-managing their BP in the next program and allow them to share their experience, then it motivates other members in the program.”—CHW.*

*“If we visit people’s homes and check their blood pressure at least once a month they will focus on diet control and exercise.”— CHW.*

*“They will definitely come to our meetings if we give them blood pressure tablets.”— CHW*.

*“The training and supportive supervision helped equip us with adequate knowledge and skills necessary to implement the intervention.” —CHW*.

## Discussion

The prevalence of high blood pressure was high but undiagnosed in the island districts of Lake Victoria in Uganda in 2022. The only factor found to be associated with high blood pressure was age. The comparison of systolic blood pressure (SBP) and diastolic blood pressure (DBP) before and after the intervention revealed significant reductions in both parameters. During the evaluation of intervention implementation, CHWs reported that the training they had received increased their skills. Supportive supervision was also identified as helpful during the program delivery. The observed reductions in both systolic and diastolic blood pressure following the intervention suggest the effectiveness of community-based approaches in managing hypertension. Furthermore, the reported increase in skills among CHWs and the importance of supportive supervision in program delivery highlight the potential of community-based initiatives to strengthen primary healthcare services.

Only 11% of participants with high blood pressure were aware that they had hypertension. This is lower than the 28% awareness reported in urban settings in Uganda [17]. This lack of awareness contributes to delayed diagnosis and treatment initiation, increasing the risk of cardiovascular complications and mortality.The high prevalence of undiagnosed high blood pressure in island districts of Lake Victoria may be due to limited access to healthcare facilities and essential medicines [4]. Most households in these island districts live beyond the targeted five kilometers maximum from the nearest health facility [5]. Health facilities are also inadequately equipped to provide screening and management of hypertension [14], and health workers are not always available in rural and remote settings [15]. More so, people in rural settings do not go for routine medical checkups and seek health services only when they are very sick [18]. Training the CHWs in monitoring BP and providing education enables a reorganization of health tasks to improve access [19].

In our study, we did not find associations between high blood pressure and some of the factors that have often been identified to be associated with high blood pressure, such as tobacco use [20] and alcohol use [21]. This may be due to differential patterns of alcohol use in remote islands compared with other settings [22]. In addition, smoking is uncommon in our setting, and our sample size may have had an impact on our ability to identify associations with smoking and high blood pressure. Our findings are consistent with a national non-communicable disease risk factor survey [23] conducted in Uganda that also found only age to be associated with high blood pressure. This implies that there must be other risk factors for high blood pressure in the Ugandan setting, other than those assessed using the WHO STEPS protocol deployed in both surveys. Indeed, obesity and diabetes were found to be associated with high blood pressure in Uganda [24]. Elsewhere, genetics have also been documented as a risk factor for high blood pressure [25]. Our baseline survey focused on identifying modifiable risk factors of high blood pressure that were targeted through lifestyle modification sessions delivered by CHWs. Addressing the high blood pressure in Uganda requires a holistic approach that acknowledges the diverse range of risk factors contributing to the condition. By implementing targeted interventions that address both modifiable and non-modifiable risk factors, public health efforts can effectively reduce the burden of high blood pressure and its associated complications in the population.

This study showed that a group-based education and monitoring program delivered by CHWs was effective in lowering blood pressure. Even though this was only a three-month intervention, the blood pressure of the participants was reduced. This underscores the potential of community-based interventions in improving hypertension management and control, particularly in regions with limited access to healthcare facilities.

In addition to the education provided to intervention participants, their blood pressure was measured at each session. This ongoing monitoring may have empowered participants to determine whether and how their lifestyle changes resulted in tangible benefits [26]. This method also provides motivation to keep or adopt new behavioral changes [26], which may be one of the intervention’s success mechanisms. This suggests that BP surveillance itself can help control BP in regions with poor access to healthcare. In addition, the program included several evidence-based components for improving blood pressure control, such as medication adherence [27], regular blood pressure monitoring [26], and encouraging lifestyle changes such as increased physical activity [28].

Our findings are consistent with other studies that showed the effectiveness of CHWs in the control of high blood pressure [29–31]. However, these studies involved intensive, time-consuming individual-based interventions. Our approach of using a group-based intervention provides a practical and potentially cheaper alternative to managing high blood pressure in hard-to reach settings. Another aspect of CHWs role in the management of high blood pressure was referring participants with high blood pressure to the clinical health workers. In communities where health-seeking behaviour is poor [18], and people only visit health facilities when they have severe symptoms, CHWs played a critical role in referring participants with high blood pressure for further clinical management. This highlights the importance of CHWs as intermediaries between communities and formal healthcare systems, facilitating timely access to clinical management for individuals with hypertension.

Qualitative data from focus groups provide an understanding of the factors that influence the causal relationship between implementation and outcome in the real world [32], and can guide scaling up the program in other settings. For example, CHWs reported that training, and supportive supervision were important enablers of intervention implementation, and participants reported that dietary changes, specifically salt reduction, and frequent blood pressure monitoring, as beneficial. These evidence-based components [33, 34], should be prioritised in the scale up of similar programs. Resident participants in the intervention program demonstrated motivation to attend meetings, citing gaps in healthcare provider guidance primarily focused on medication prescriptions. This highlights the need for healthcare systems to prioritize holistic management strategies for chronic conditions like hypertension, focusing on lifestyle modifications alongside medication prescriptions. Additionally, the cost emerged as a significant barrier to BP control, indicating the need for interventions that address medication affordability to ensure equitable access to essential medications. Although CHWs are highly regarded within their communities for the support and care they provide, they face challenges, particularly related to transportation and mobility issues, which impact their ability to reach households effectively. Ensuring adequate resources and support for CHWs, including transportation assistance, could strengthen their ability to reach households effectively and deliver interventions successfully.

### Study strength

The study has strengths. it introduces a novel approach by utilizing CHWs for blood pressure screening and management, which had not been done previously in Uganda. The approach fosters community engagement and participation, promoting ownership of healthcare initiatives within the community.

### Study limitations

There are some limitations to our study that may influence the interpretation of our findings. First, the 3-month intervention was relatively short. Because of this, we cannot determine whether there is a long-lasting improvement in controlling high blood pressure from our group-based education and monitoring program. Second, some of the observed reduction in BP may be attributable to the Hawthorne effect, whereby participants alter behaviors just because they are being observed, and do not sustain it long term.Third, many of the diagnosed participants did not come for follow-up. Additionally, the absence of a comparison group limits the ability to establish effectiveness definitively. The motivation for this approach was to ensure equitable access to potentially beneficial intervention while still allowing for rigorous evaluation. The design was also resource-efficient, we needed to widen the geographic scope with the intervention since the island districts had limited access to health facilities and community health workers were not conducting screening and intervention for hypertension.

## Conclusions

High blood pressure was common but undiagnosed in residents of the island districts of Lake Victoria. The CHW-led group-based self-management and education for improving the control of high blood pressure was effective in reducing both SBP and DBP and is potentially feasible. There is considerable potential to scale up across rural Uganda and potentially other resource-limited regions in other countries.

## Data Availability

The datasets that supported the findings of this project belong to the Training Programs in Epidemiology and Public Health Interventions Network (TEPHINET) and Uganda Public Health Fellowship Program (UPHFP). The datasets used and/or analyzed for the current study are available from the corresponding author on reasonable request and with and with permission from TEPHINET and UPHFP.
